# Changes in Capsular Serotype Alter the Surface Exposure of Pneumococcal Adhesins and Impact Virulence

**DOI:** 10.1371/journal.pone.0026587

**Published:** 2011-10-19

**Authors:** Carlos J. Sanchez, Cecilia A. Hinojosa, Pooja Shivshankar, Catherine Hyams, Emilie Camberlein, Jeremy S. Brown, Carlos J. Orihuela

**Affiliations:** 1 Department of Microbiology and Immunology, The University of Texas Health Science Center, San Antonio, Texas, United States of America; 2 Department of Medicine, Centre for Respiratory Research, Royal Free and University College Medical School, Rayne Institute, London, United Kingdom; Instituto Butantan, Brazil

## Abstract

We examined the contribution of serotype on *Streptococcus pneumoniae* adhesion and virulence during respiratory tract infection using a panel of isogenic TIGR4 (serotype 4) mutants expressing the capsule types 6A (+6A), 7F (+7F) and 23F (+23F) as well as a deleted and restored serotype 4 (+4) control strain. Immunoblots, bacterial capture assays with immobilized antibody, and measurement of mean fluorescent intensity by flow cytometry following incubation of bacteria with antibody, all determined that the surface accessibility, but not total protein levels, of the virulence determinants Pneumococcal surface protein A (PspA), Choline binding protein A (CbpA), and Pneumococcal serine-rich repeat protein (PsrP) changed with serotype. *In vitro*, bacterial adhesion to Detroit 562 pharyngeal or A549 lung epithelial cells was modestly but significantly altered for +6A, +7F and +23F. In a mouse model of nasopharyngeal colonization, the number of +6A, +7F, and +23F pneumococci in the nasopharynx was reduced 10 to 100-fold versus +4; notably, only mice challenged with +4 developed bacteremia. Intratracheal challenge of mice confirmed that capsule switch strains were highly attenuated for virulence. Compared to +4, the +6A, +7F, and +23F strains were rapidly cleared from the lungs and were not detected in the blood. In mice challenged intraperitoneally, a marked reduction in bacterial blood titers was observed for those challenged with +6A and +7F versus +4 and +23F was undetectable. These findings show that serotype impacts the accessibility of surface adhesins and, in particular, affects virulence within the respiratory tract. They highlight the complex interplay between capsule and protein virulence determinants.

## Introduction

Capsular polysaccharide (capsule) is the most important virulence determinant of *Streptococcus pneumoniae*, surrounding and protecting the bacterium against complement-mediated phagocytosis and bacterial killing by extracellular traps [1], [2], [3], [4]. The requirement for capsule is made evident by the fact that all invasive clinical isolates of *S. pneumoniae* are encapsulated [5], unencapsulated pneumococci are incapable of causing pneumonia or sepsis in animal models [6], [7], and antibodies against capsule promote opsonization and confer protection [8], [9]. Indeed, purified capsule serves as the protective antigen in all vaccines approved worldwide against *S. pneumoniae*.

Although capsule affords protection against host-clearance, it also hinders the ability of the pneumococcus to adhere to host epithelial cells [10], [11]. This is likely due to a blocking of bacterial adhesins by the capsule that prevents their interaction with host ligands. To compensate, the pneumococcus spontaneously undergoes phase variation, alternating between a transparent low-capsule phenotype which is adhesive to host cells but subject to opsonophagocytosis, and an opaque high-capsule phenotype that is resistant to opsonophagocytosis but less able to attach to cells [12]. Additionally, some pneumococci carry Pneumococcal serine-rich repeat protein (PsrP), a member of the serine-rich repeat protein family, or pili, that are thought to extend outward through the capsule and mediate adhesion even in the presence of capsule [13], [14], [15], [16], [17]. Finally, studies using scanning electron microscopy suggest that the pneumococcus actively down-regulates capsule production when attached to epithelial cells [11]. Thus, the presence of capsule profoundly affects how the pneumococcus interacts with host epithelial cells and not just phagocytes.

To date more than 90 chemically and serologically and distinct capsule types of *S. pneumoniae* have been described. Importantly, not all serotypes are equally capable of colonizing the nasopharynx or causing invasive disease. For example, in the United States only 13 serotypes (i.e. 1, 3, 4, 5, 6A, 6B, 7F, 9V, 14, 18C, 19A, 19F, and 23F) are responsible for 80–90%of invasive pneumococcal infections [18], [19], [20]. The reasons for why some serotypes are more likely to cause disease remain unclear, as pneumococci are genetically diverse and non-invasive clones belonging to these disease-associated capsule types have also been described [21], [22]. Thus the only way to assess the individual contribution of capsule type to virulence is to compare isogenic capsule-switched strains of the same genetic background.

In 1994, studies by Kelly *et al.* showed that isogenic changes in capsule type unpredictably altered virulence following intraperitoneal challenge. Replacement of capsule type 5 on a highly virulent strain with type 3 resulted in a complete loss of virulence. In contrast, change of a non-virulent serotype 6A strain to capsule type 3 enhanced virulence [23]. One proposed explanation for this was that capsule type affected resistance to both complement deposition and opsophagocytic uptake. In support of this notion, recent studies have shown that the switching of a serotype 4 isolate to serotype 23F or 6A increased C3b deposition and phagocytic uptake, whereas replacement with 7F had a minimal effect [24]; similar results have been reported by Melin *et al*. [25], [26], [27]. Importantly, differences in C3b deposition on the bacterial surface have been suggested to be due, in part, to differences in the surface exposure of pneumococcal proteins with anti-complement activity such as Choline binding protein A (CbpA), which binds to Factor H and is an adhesin for laminin receptor and the polymeric immunoglobulin receptor [28], [29], [30], and Pneumococcal surface protein A (PspA), which inhibits complement deposition by unknown mechanisms [31]. For example, replacement of strain D39 serotype 2 capsule with serotype 3 reduced the reactivity of antisera against PspA [32]. Thus, interplay between capsule and surface exposed virulence determinants most likely occurs and impacts virulence potential.

Herein, using isogenic mutants, we tested whether capsule type also impacts pneumococcal adhesion and virulence at sites where bacterial attachment is required. We determined that changes in capsule altered surface accessibility of the pneumococcal adhesins PsrP and CbpA as well as PspA, modulated bacterial adhesion *in vitro*, and attenuated nasopharyngeal colonization and virulence within the lungs. These studies provide insight into the complex interplay between capsule and bacterial surface components and help explain why not all combinations are compatible for virulence.

## Methods

### Bacterial strains and tissue culture cell lines used

The isogenic capsule switch opaque phase variant mutants were kindly provided as a gift by Dr. Jeffery Weiser at The University of Pennsylvania and were generated using a Janus cassette [24]. *S. pneumoniae* serotype 4, strain TIGR4 was used as the background strain [21], [33]. The capsule switch strains include TIGR4 derivatives expressing the 6A (+6A), 7F (+7F), and 23F (+23F) capsule type as well as a deleted and restored capsule type 4 (+4). The latter served as the control for the panel of isogenic mutants. Previously, we have shown that +6A, +7F, and +23F carry equivalent amounts of capsule as +4 and have comparable growth rates in Todd Hewitt Broth (THB) and serum [24]. An unencapsulated derivative of TIGR4, T4R, was also used as a negative control [34]. Unless otherwise indicated, pneumococci were grown on tryptic soy blood agar plates (Remel, USA) or THB at 37°C in 5%CO_2_.

### Confirmation of appropriate capsule production by switch mutants

Multiplex PCR using genomic DNA as template and serotype specific primers was performed to verify the presence of specific capsule type genes in each strain [35], [36]. Production of the correct polysaccharide capsule was confirmed by agglutination using type-specific antibodies (Statens Serum Institut, Denmark).

### Assessment of surface accessibility of the pneumococcal adhesins on antibody- immobilized surfaces

Polystyrene 24-well plates were coated with rabbit polyclonal antibodies against CbpA and PsrP (1∶250 in PBS) overnight. Serum from a naïve animal was used as a negative control. Bacterial cultures were diluted in phosphate buffered saline (PBS) to an OD_620_ of 0.1, and incubated for 1 h at 37°C on the antibody coated wells. Following incubation, wells were gently washed 3 times with PBS to remove unbound bacteria and attached bacteria were freed with gentle scraping. Bacterial adhesion was determined by addition of 100 µl PBS, plating of the bacterial suspension, and extrapolation from colony counts following overnight incubation. Each experiment contained at least 3 biological replicates for each strain tested.

### Detection of surface expression of pneumococcal proteins by flow cytometry

Indirect immunofluorescence was carried out to determine the ability of antibodies against recombinant CbpA, PsrP and PspA to bind to the surface of intact *S. pneumoniae* (17). Antibodies against PsrP were previously generated [13], [37], whereas those against CbpA where a kind gift from Dr. Elaine Tuomanen (Memphis, TN). Antibodies against PspA were obtained from Santa Cruz Biotechnology (sc-17483, Santa Cruz, CA). Confirmed opaque pneumococci were harvested directly from blood agar plates grown overnight or from exponential growth phase liquid cultures (Optical Density [OD]_620_ = 0.5), washed in sterile PBS and suspended in staining buffer (0.05% sodium azide and 1% bovine serum albumin in PBS). Approximately 2×10^7^ bacteria were incubated with 10% rabbit antisera to CbpA or PsrP or with 10% goat antiserum to PspA. As a control rabbit and goat IgG isotypes were used (Sigma Aldrich, St. Louis MO). Following incubation at 4°C for 1 h, bacteria were washed and incubated with a 1∶100 dilution of a F(ab')2 fragment of goat anti-rabbit IgG (H+L) or donkey anti-goat conjugated to Phycoerythrin (Jackson Immunoresearch Laboratories, Westgrove PA). Bacteria were washed in PBS and subjected to flow cytometry using a Becton Dickinson LSR-II flow cytometer using forwards and side scatter parameters to gate on at least 10,000 bacteria. Results were analyzed using FlowJo v9.3 software (Becton Dickinson) and the data is presented as mean fluorescence intensity (MFI) and as the percent of the bacterial population positive for relative markers.

### Bacterial Adhesion Assays

A549 (human lung alveolar epithelium cell line; ATCC CRL-185) and Detroit 562 (human nasopharyngeal epithelial cells; ATCC CCL-138) were maintained in either F-12 media supplemented with 10% fetal bovine serum or MEM media supplemented with 10% FBS, 10% lactoalbumin hydrolate, and 2 mM L-glutamine, respectively. All cell lines were maintained at 37°C in 5% CO_2_. Adhesion assays were performed as previously described [37]. Cells were grown to 90–95% confluence (∼10^6^ cells/well) in 24-well tissue culture polystyrene plates. Cells were washed then exposed to media containing 10^7^ CFU/ml of pneumococci for 1 h at 37°C in 5% CO_2_. Non-adherent bacteria were removed by washing the cells 3 times with PBS. Adherent bacteria were quantitated by lysis of the cell monolayer with 0.1% Triton X-100 in PBS, serial dilution of the lysate followed by plating on blood agar plates, and extrapolation from colony counts the next day. Each experiment contained at least 3 biological replicates for each strain tested.

### Virulence studies in mice

Challenge experiments described below were performed at the University of Texas Health Science Center at San Antonio using an Institutional Animal Care and Use Committee approved protocol 09022-34. To minimize distress mice were anesthetized during experimental challenge and prior to euthanasia. Five-week-old, female, BALB/cJ mice (The Jackson Laboratories, Bar Harbor, ME) were anesthetized with 2.5% vaporized isoflurane. We have previously used BALB/c mice to determine the anatomical site-specific contribution of numerous pneumococcal virulence determinants including PsrP and CbpA [30], [37], [38].

The intranasal, intratracheal, and intraperitoneal challenge models have been previously described [37]. For intranasal challenge, anesthetized mice were held upright and 10^6^ CFU of *S. pneumoniae* in 25 µl PBS was administered into the left nostril in a drop-wise fashion. For intratracheal challenge, anesthetized mice were hung upright by their incisors and 10^5^ CFU of *S. pneumoniae* in 100 µL of PBS was placed at the back of the throat. Forced aspiration was induced by gently pulling the tongue outward and covering the nostrils. For intraperitoneal challenge mice were injected with 10^4^ CFU in 100 µl PBS. On designated days, nasopharyngeal lavage with 10 µl saline or collection of blood from the tail vein was performed. Nasopharyngeal lavage was performed by drop-wise instillation of PBS and recovery from the same nostril after a few seconds. Bacterial titers in the nasopharynx and blood, respectively, were determined by serial dilution, plating, and extrapolation from colony counts the next day. For determination of bacterial titers in the lungs, mice were sacrificed, the lungs excised, weighed, and homogenized in PBS. Bacterial burden in the lungs was assessed per gram of homogenized tissue.

Studies with isolated alveolar macrophages were performed at University College London under Home Office license PPL70/6510. Animal experiments were also approved by the University College London Biological Service Unit Ethics Committee. Sex-matched 6 week old CD1 mice were inoculated intranasally under halothane anesthesia with 10^7^ CFU FAM-SE labeled *S. pneumoniae*. Mice were sacrificed after 4 hours and bronchoalveolar lavage fluid (BALF) collected using an angiocatheter and flushing of the lungs with 1 ml sterile PBS (35). Bacterial CFU were calculated by plating serial dilutions of BALF. *S. pneumoniae* phagocytosis by BALF AMs was analyzed using a FacsCalibur (Becton Dickinson) to identify the proportion of cells associated with fluorescent bacteria.

### Statistical analysis

For statistical comparisons between 2 cohorts a two-tailed un-paired Student's *t*-test was used. For comparisons between multiple cohorts a One-Way ANOVA or Dunn's Multiple Comparison test was used. Statistical analyses were performed using SigmaStat software (Systat Software, Chicago IL).

## Results

### Levels of CbpA, PsrP and PspA were unaffected by changes in serotype

**Figure 1:**
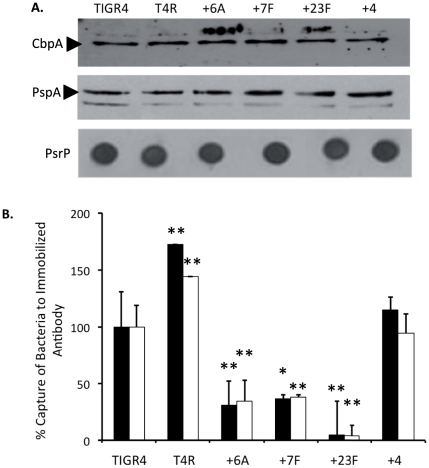
Differences in surface expression of pneumococcal ligands is dependent on serotype. **A**) Immunoblot analysis comparing relative protein levels in whole cell lysates of TIGR4, an unencapsulated derivate (T4R), and the panel of capsular switch mutants. Whole cell lysates (10 µg) were separated by 12%SDS-PAGE and transferred to nitrocellulose or directly blotted onto membranes and probed with antisera to choline binding protein (CbpA), pneumococcal surface protein A (PspA), or the pneumococcal serine-rich repeat protein (PsrP). **B**) Capture of bacteria to immobilized antibodies to CbpA (black bars) and PsrP (white bars). TIGR4 and the panel of isogenic mutants were incubated in wells pre-coated with serum to CbpA or PsrP and surface expression was indicated by the amount of bacteria captured to immobilized antibody. Values are presented as a percentage of capture of the TIGR4 strain. Statistical analysis was performed using a two-tailed Student's *t*-test versus TIGR4. Single asterisk indicates *P<*0.05, double asterisks indicates *P<0.001*.

Prior to experimentation, +6A, +7F, +23F and +4 were confirmed to produce the appropriate capsule type by multiplex PCR for serotype specific genes as well as agglutination with serotype specific antiserum (Figure S1). Additionally, we assessed whether switching capsule impacted production of proteins. As determined by immunoblot analysis, +6A, +7F, +23F, had equivalent total levels of CbpA, which binds to Laminin receptor and Polymeric immunoglobulin receptor [29], [30], PsrP, which binds to Keratin 10 [13], and PspA, which inhibits complement deposition [18], [31], [39], as +4, TIGR4, and T4R, an unencapsulated TIGR4 derivative (Figure 1A).

### Surface accessibility of pneumococcal ligands is dependent on serotype

Using polystyrene plates coated with antibody specific for either CbpA or PsrP, we first determined that +6A, +7F, and +23F had a dramatic reduction in their ability to be captured on a flat surface versus +4. In contrast, +4 was captured at levels equivalent to TIGR4, and T4R showed enhanced retention (Figure 1B). As the antibody was immobilized, and therefore unable to penetrate the capsule layer, this experiment modeled the ability of these adhesins to bind to their ligand on the host cell surface.

**Figure 2:**
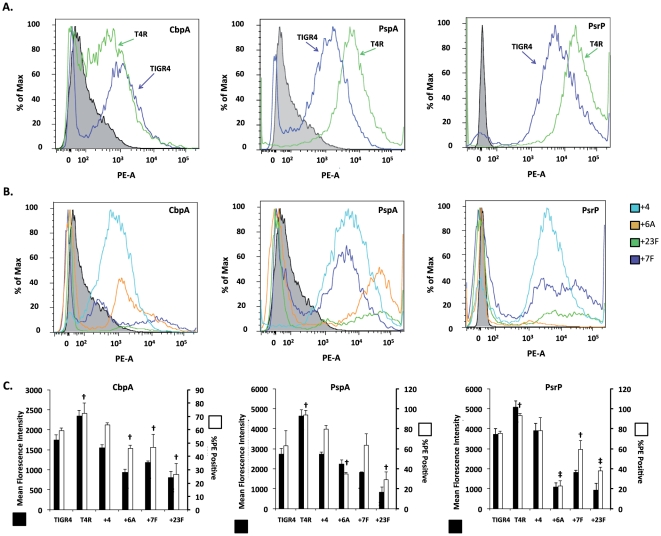
Effect of serotype and surface accessibility of pneumococcal ligands. Flow cytometry histograms for pneumococcal surface ligands CbpA, PspA, and PsrP in **A**) TIGR4 and its unencapsulated derivative (T4R) and **B**) the isogenic capsular switch strains +4, +6A, +7F, +23F. Histograms are representative of three independent experiments using bacteria collected from overnight plates (similar results were obtained using planktonic pneumococci). Surface accessibility of CbpA, PspA, and PsrP as measured by mean fluorescence intensity (MFI; black bars) and %PE+ positively labeled cells (white bars). Results from three independent experiments are shown. Statistical analysis was performed using a two-tailed Student's *t*-test versus T4. Single cross indicates *P<*0.05, double cross indicates *P<0.001*.

We also compared the ability of free labeled-antibody to bind to CbpA, PsrP, and PspA on the bacterial surface using flow cytometry. Compared to TIGR4, T4R had significantly increased mean fluorescent intensity (MFI) demonstrating that capsule blocked antibody access to these surface proteins (Figure 2A, C). Compared to +4, the capsule switch strains +6A, +7F, and +23F also had reduced accessibility to antibody as determined by MFI and the detected percentage of PE+ cells (Figure 2B,C). This remained constant whether bacteria were collected from overnight blood agar plates (Figure 2) or from logarithmic growth phase liquid cultures (data not shown). Thus, changes in serotype reduced surface accessibility of CbpA, PsrP and PspA on the bacteria outer surface to immobilized and free antibody.

### Effect of capsule type on bacterial adhesion *in vitro*

**Figure 3:**
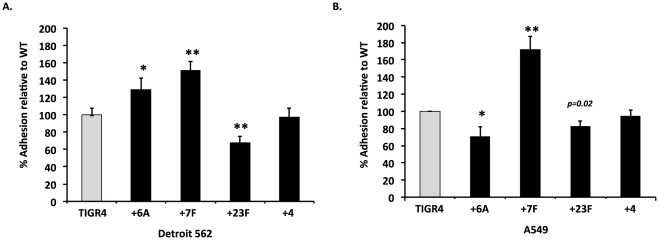
Effect of serotype on bacterial adhesion. *In vitro* adhesion assays measuring the ability of TIGR4 or the isogenic capsular switch mutants to adhere to **A**) human lung alveolar (A549) and **B**) nasopharyngeal (Detroit) cell lines. Adhesion is expressed as a percentage relative to TIGR4 adhesion. Statistical analysis was performed using a two-tailed Student's *t*-test versus TIGR4. Single asterisk indicates *P<*0.05, double asterisks indicates *P<0.001*.

To assess whether changes in surface accessibility of CbpA and PsrP altered bacterial adhesion *in vitro*, we examined the ability of the capsule switch strains to adhere to Detroit 562 cells, a human nasopharyngeal cell line, and A549 cells, a type II pneumocyte cell line (Figure 3). Strain +4, the capsule switch control strain, adhered to monolayers of both cell lines at levels equivalent to TIGR4. In contrast, +6A adhered to Detroit 562 and A549 cells at levels slightly greater and lower than +4, respectively. +7F demonstrated a moderately enhanced capacity to adhere to these cells; a 1.5 and 1.7-fold increase in adhesion was observed versus +4, respectively. Finally, +23F had a less than 2-fold reduction in its ability to adhere to Detroit 562 cells but not A549 cells. Thus changes in capsule type resulted in unexceptional but reproducible changes in bacterial adhesion *in vitro* that were capsule type and host cell type dependent.

### Capsule type affects nasopharyngeal colonization and virulence potential

**Figure 4:**
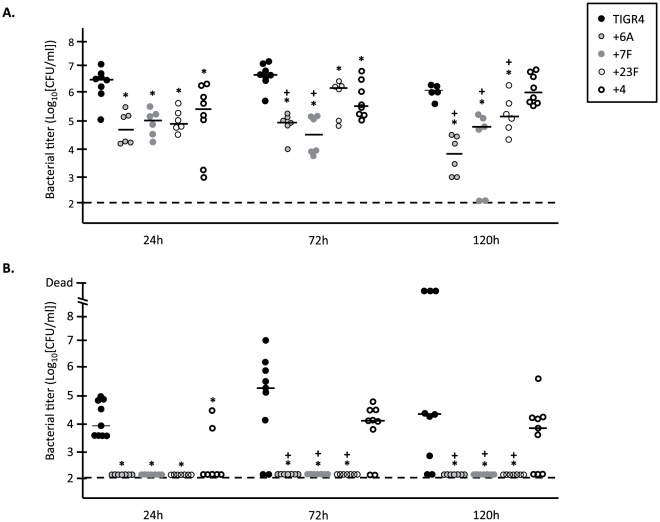
Genetic switch of serotype alters nasopharyngeal colonization and virulence in mice. Bacterial titers were measured for individual 5-week old female BALB/cJ mice intranasally infected with 10^6^ CFU of the parental TIGR4 WT (n = 8) or the isogenic capsular switch strains +6A, +7F, +23F, and +4 (n = 6). **A**) Nasal lavages and **B**) blood were collected at 24, 72, and 120 h post-infection. Horizontal bars represent the median value. Statistical analysis was performed using One-Way ANOVA in comparison to both the TIGR4 strain (*) and the internal +4 control strain (+). Asterisk and the cross indicate a significance value of *P*<0.05.

Having observed discrepant results in regards to the impact of serotype switch on bacterial antibody capture and cell adhesion, we tested the effect of changing capsule type on nasopharyngeal colonization and subsequent development of pneumonia; infectious disease states that have previously been shown to require CbpA and PsrP [37], [38]. Importantly, the +4 control strain was found to be modestly attenuated compared to the TIGR4 strain in the intransal and intraperitoneal experiments (Figure 4A, 5B). Thus, insertion of the Janus cassette had inadvertent consequences and only comparisons between the +6A, +7F, and +23F versus +4 are valid.

Following intranasal challenge, +6A, +7F, and +23F had significantly reduced median bacterial titers in the nasopharynx versus +4 as early as 24 h post-infection and continuing up to 120 h (Figure 4A). For +6A a 100-fold reduction in median bacterial titers was observed after 120 h. Surprisingly, only +4 caused bacteremia following intranasal challenge (Figure 4B). At no time were +6A, +7F, or +23F detected in the bloodstream of challenged mice. Thus the capsule switch strains were moderately attenuated in their ability to colonize the nasopharynx but completely unable to cause invasive disease following intranasal challenge.

**Figure 5:**
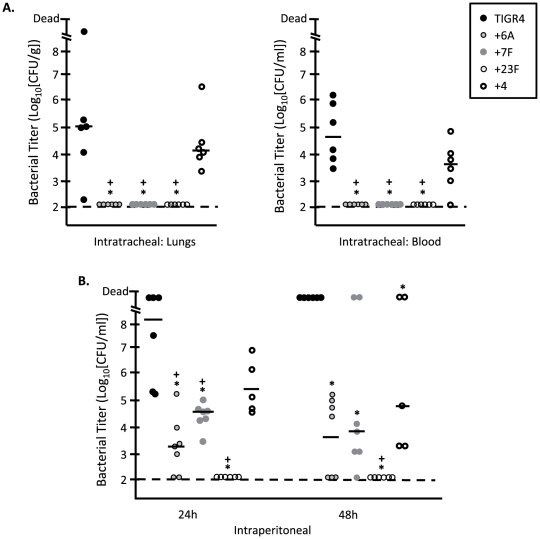
Serotype affects virulence in the lungs. Bacterial titers were measured for individual 5-week old female BALB/cJ mice infected via **A**) intratracheal (10^5^ CFU) or **B**) intraperitoneal (10^4^ CFU) routes with TIGR4 WT (n = 6) or the isogenic capsular switch strains +6A, +7F, +23F, and +4 (n = 6). For intratracheal challenge samples were collected at 48 h post-infection, and for intraperitoneal challenge samples were collected at 24 and 48 hours. Horizontal bars represent the median value. Statistical analysis was performed using One-Way ANOVA in comparison to both the TIGR4 strain (*) and the internal +4 control strain (+). Asterisk and the cross indicate a significance value of *P*<0.05.

To dissect how capsule type might be affecting disease progression, we subsequently infected mice intratracheally and intraperitoneally (Figure 5). Mice infected intratracheally with 10^5^ CFU of the +6A, +7F, and +23F capsule switch strains had no bacteria in their lungs or blood at any time tested. In contrast, mice infected with +4 strain developed low-grade pneumonia and bacteremia that was comparable to wild type (Figure 5A). Following intraperitoneal challenge, +6A, +7F were capable of persisting within the blood at lower levels than +4, whereas +23F was immediately cleared (Figure 5B). Thus, changes in capsule type had a strong attenuating affect within the lungs that precluded entry of +6A, +7F into the bloodstream, and +23F was attenuated in all anatomical sites tested.

### Capsule switch strains are readily cleared by the host

**Figure 6:**
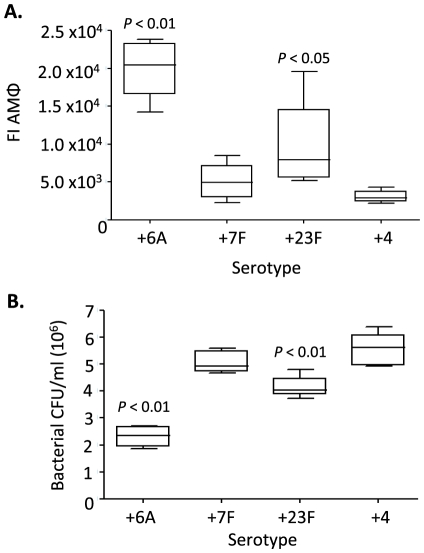
Clearance of FAMSE-labeled capsule switch strains from the lungs and uptake by alveolar macrophages 4 hours-post inoculation. **A**) Alveolar macrophage phagocytosis of CPS switch strains as determined by FACS. Data presented as the median (IQR) fluorescence index (percentage of fluorescent macrophages multiplied by the geo mean MFI) for BALF macrophages (n = 5). **B**) Median bacterial titers in bronchoalveolar lavage fluid (BALF) (n = 5). For panels A and B samples were collected 4 hours after intranasal challenge with 5×10^6^ CFU FAMSE-labeled CFU, and the *P* values given above the respective box and whisker plot for individual strains are for comparison to the +4 strain. A Dunn's Multiple Comparison test was used for statistical analyses.

Within the lungs, establishment of pneumococcal pneumonia is dependent on both the ability of the bacteria to attach to cells, yet remain resistant to phagocytosis by resident alveolar macrophages. To investigate whether effects on macrophage-mediated immunity might also explain the strong attenuation of the capsule switch strains in the lungs, mice were inoculated with fluorescent *S. pneumoniae* strains and bacterial uptake by alveolar macrophages assessed 4 hours after inoculation using flow cytometry. The association of alveolar macrophages with +6A and +23F strains was increased compared to the +4 strain, whereas the association of the +7F strain was similar (Figure 6A). Bacterial CFU in the BALF was consistent with these results, with significantly fewer bacterial CFU for the +6A and +23F but not the +7F compared to +4 in BALF 4 hours after inoculation (Figure 6B). Hence, serotype 6A and 23F increased uptake of *S. pneumoniae* by alveolar macrophages and the rate of bacterial clearance from the lung, whereas capsule type 7F had no effect.

## Discussion

While considerable work has been done to examine the effect of capsule type on complement deposition and the effect of serotype on the ability of pneumococci to colonize the nasopharynx and survive in bloodstream [4], [24], [26], [27], [28], [40], [41], this is the first study to assess the impact of capsule type on bacterial adhesin function and the development of pneumonia. Given that pneumonia is the most common serious disease presentation caused by the pneumococcus and host-cell adhesion is a requisite event for development of invasive disease [15], [29], [37], [38], [42], our study begins to address this important lapse in our understanding of *S. pneumoniae* pathogenesis.

The most studied feature of the pneumococcal capsule is its inhibitory effect on opsonization and phagocytosis. In regards to the latter, using the same isogenic capsule switch strains, we and others have shown that capsule type impacts the amount of C3b that is deposited on the bacteria surface and subsequent uptake by neutrophils [4], [24], [25], [26], [27]. Our current findings suggest that the capsule type might also impact other pathogenic mechanisms, in particular PsrP and CbpA function. In support of this notion, we observed reduced capture of intact bacteria by immobilized antibody against PsrP and CbpA in the +6A, +7F, and +23F capsule switch strains versus +4, altered accessibility of free-antibody to these adhesins and to PspA by flow cytometry, and a stronger attenuation for +6A and +7F in the lungs versus the blood. In the lungs the pneumococcus requires both bacterial attachment and resistance to opsonophagocytosis in order to persist. The combination of these two factors may explain the different effects of serotype on virulence between pneumonia and sepsis models.

Pneumococcal adhesion is dependent on the ability of bacterial proteins to interact with ligands on the host cell surface. The presence of capsule is known to negatively impact these interactions, thus investigators routinely use unencapsulated pneumococci when examining the role of their selected adhesins [42], [43], [44]. Our *in vitro* finding that capsule type impacted the ability of immobilized antibody against CbpA and PsrP to capture pneumococci suggests that the biochemical properties of these capsule types alters their exposure on the outer surface of the pneumococcus. Results from our cytometric analyses support this notion and showed that the presence of capsule decreased the ability of free antibody against PspA, CbpA, and PsrP to gain access to these proteins, and that capsule types 6A, 7F, and 23F permitted less accessibility that capsule type 4. Notably, PsrP and CbpA deficient mutants have been demonstrated to be more severely attenuated in the lungs rather than bloodstream of experimentally infected mice [37], [38]. Thus, the observed impact of diminished CbpA and PsrP exposure by +6A, +7F, and +23F versus +4, is consistent with past and present experimental observations *in vivo*.

Importantly, the modest reduction observed for only +23F in the *in vitro* bacterial adhesion assays is difficult to reconcile with our data showing reduced surface accessibility of CbpA and PsrP. This discrepancy may be because the pneumococcus uses multiple adhesins to attach to host-cells and other unaffected adhesins compensated. For example, the pneumococcus also binds to epithelial cells through phosphorylcholine residues on its cell wall, with the tip of a pilus structure, using the MSCRAMMs (Microbial Surface Components Recognizing Adhesive Matrix Molecules) PavA and PavB, and with a multitude of normally cytoplasmic enzymes that decorate the bacterial surface, among others [45], [46], [47]. If this compensatory effect were to hold true *in vivo*, the observed dramatic attenuation of the isogenic switch strains in the lungs would therefore have to be the result of capsule-mediated changes in the secondary function of surface proteins. While we did not explore this possibility, CbpA has been shown to bind to Factor H and further inhibit complement deposition [48]. Likewise, PsrP also mediates *in vivo* biofilm formation [49]. Thus altered function on top of reduced accessibility may explain the discrepancy between cell adhesion assays and virulence.

CbpA, PspA, and PsrP are highly variable and show strong associations with clonotype and/or capsular type. For example numerous isotypes of CbpA exists with alterations in the R1 and R2 domains of the protein [50]. Similarly, the protein PspA is classified into two major families depending on its amino acid composition [31]. Sequence analysis of the published genomes indicates that PsrP also demonstrates considerable variability, in particular the length of its SRR2 domain that serves to extend the protein outward [13]. The observed difference in the surface accessibility of these proteins and their known structural variability raises the possibility that some versions of CbpA and PsrP might only be compatible with certain serotypes. Perhaps invasive clones of each serotype carry the optimized version of these proteins for the corresponding serotype. This would perhaps help explain why certain virulence determinants such as PsrP are not equally distributed among serotypes [17]. Interestingly, in the MFI experiments with the capsule switch strains and free antibody (Figure 2B), we observed that some of the graphs had what might be construed as two distinct cell populations (e.g. PspA). As we used confirmed opaque bacteria, we could exclude phase-variation as the cause of this bimodality. Furthermore, as we saw similar results following experiments with overnight cultures and planktonic cultures, we could exclude the presence of dead cells. The cause for this phenomena remains unknown, albeit for the pneumococcal pilus a non-phase variable bimodilty has been recently described [51].

Finally, we have previously demonstrated that complement activity was maximum against +6A and +23F, followed by +7F, and then +4 [24]; and this was reflected in our current experiments examining bacterial uptake by resident alveolar macrophages in the lungs, measurement of bacterial titers in the bronchoalveolar lavage fluid, and after intraperitoneal inoculation where mice challenged with +6A, +7F, and +23F had significantly lower numbers of pneumococci in their blood than those challenged with +4. Thus any effects of capsule on CbpA and PsrP function are concomitant with those that occur in regards to opsonophagocytosis.

Importantly, all the strains used in this study were of the opaque phase variation, had similar capsule thickness, and showed equivalent growth rates *in vitro* [24]. While we were surprised to observe that the +4 strain was attenuated *in vivo* in comparison to wild type TIGR4, suggesting an inadvertent affect of the Janus insertion or another mutation incurred during creation, the fact that we only compared +6A, +7F, +23F to +4 in these studies allowed for its control. Thus the observed differences can be attributed to the biological properties of the serotype and not the relative fitness or amount of capsule surrounding the bacterium.

Taken together our findings indicate that there is complex relationship between capsular and non-capsular determinants that synergistically effect virulence potential, particularly in the lungs. Based on our findings and those of other investigators, we propose that the capsular type imposes a biochemical threshold for surface accessibility/functionality of proteins which must be overcome by altered or enhanced expression of other genetic determinants. As a result, certain genetic backgrounds (i.e. clonotypes) are better suited for certain serotypes versus others. We speculate that this explains why certain forms of proteins are expressed in specific capsular type backgrounds, moreover, why we observed that replacement of capsular type can have a profound effect on virulence independent of strain background.

## Supporting Information

Figure S1 **Confirmation of expression of capsule types by the capsular switch strains. A)** Appropriate capsular serotype production was confirmed for the switch strains by a series of five different PCR reactions which amplified serotype-specific genes for capsule type 4, 6A, 7F, and 23F as well as *cpsA* (positive control). **B)** Agglutination assay using serotype specific antiserum confirmed production of capsule types by the capsule switch mutants.
(EPS)
